# Working memory load modulates the processing of audiovisual distractors: A behavioral and event-related potentials study

**DOI:** 10.3389/fnint.2023.1120668

**Published:** 2023-02-23

**Authors:** Yichen Yuan, Xiang He, Zhenzhu Yue

**Affiliations:** Department of Psychology, Sun Yat-sen University, Guangzhou, China

**Keywords:** multisensory integration, audiovisual, distractor, working memory load, n-back task

## Abstract

The interplay between different modalities can help to perceive stimuli more effectively. However, very few studies have focused on how multisensory distractors affect task performance. By adopting behavioral and event-related potentials (ERPs) techniques, the present study examined whether multisensory audiovisual distractors could attract attention more effectively than unisensory distractors. Moreover, we explored whether such a process was modulated by working memory load. Across three experiments, n-back tasks (1-back and 2-back) were adopted with peripheral auditory, visual, or audiovisual distractors. Visual and auditory distractors were white discs and pure tones (Experiments 1 and 2), pictures and sounds of animals (Experiment 3), respectively. Behavioral results in Experiment 1 showed a significant interference effect under high working memory load but not under low load condition. The responses to central letters with audiovisual distractors were significantly slower than those to letters without distractors, while no significant difference was found between unisensory distractor and without distractor conditions. Similarly, ERP results in Experiments 2 and 3 showed that there existed an integration only under high load condition. That is, an early integration for simple audiovisual distractors (240–340 ms) and a late integration for complex audiovisual distractors (440–600 ms). These findings suggest that multisensory distractors can be integrated and effectively attract attention away from the main task, i.e., interference effect. Moreover, this effect is pronounced only under high working memory load condition.

## 1 Introduction

In our daily lives, we are surrounded by information from different senses, such as audition, vision, touch, and so on. Due to the limited capacity of attention, we cannot process all the information effectively. Previous studies have demonstrated that multisensory stimuli can be integrated and capture attention more effectively than unisensory stimuli (Santangelo and Spence, 2007). For example, multisensory processing shows a clear processing advantage over unisensory processing (ten Oever et al., 2016), yielding more precise representation (Frassinetti et al., 2002), more accurate localization (Van der Stoep et al., 2014), and enhanced stimulus detection (Diederich and Colonius, 2004).

In the past decades, multisensory integration has been extensively investigated by many researchers. However, whether attention is needed for multisensory integration is still a controversial question. Some studies have found that multisensory integration can occur pre-attentively (Caclin et al., 2002; Santangelo and Spence, 2007; Zimmer and Macaluso, 2007; Santangelo et al., 2008a; Van der Burg et al., 2008, 2009; Wahn et al., 2017). For instance, Santangelo and Spence (2007) and Santangelo et al. (2008a) investigated attention capture by unisensory or multisensory cues under different perceptual loads. In their spatial cueing task, a visual target was presented after a unisensory (visual or auditory) or multisensory (audiovisual) cue. Participants were required to discriminate the location of the target under low load (no secondary task) or high load conditions (with a concurrent rapid serial visual presentation task). Their results showed that both unisensory and multisensory cues captured attention in the low load condition; whereas only multisensory cues could capture attention in the high load condition. These findings suggest that the multisensory stimuli can be integrated pre-attentively, thus capturing attention more effectively than unisensory stimuli, especially under high perceptual load condition. Similarly, Van der Burg et al. (2008) adopted a visual search task to examine the influence of perceptual load on multisensory integration. They found that under both low and high perceptual load conditions, the target “popped out” when the visual target was presented concurrently with an auditory pip, suggesting that visual and auditory stimuli were integrated and captured attention effectively irrespective of perceptual load.

However, other studies have shown that multisensory integration can be modulated by attention (Alsius et al., 2005, 2014; Talsma and Woldorff, 2005; Talsma et al., 2007; Hyun et al., 2009; Gibney et al., 2017; Lunn et al., 2019). For instance, Lunn et al. (2019) investigated the modulation of perceptual load on attention capture by multisensory stimuli. Participants were required to search for the visual targets in the central display or to indicate the location of the visual or audiovisual targets in the periphery display. The visual search display consisted of one target letter and other different letters in the high load condition, whereas one target letter and small placeholder “O”s were included in the display in the low load condition. The multisensory integration was observed in the high load condition but not in the low load condition, indicating that multisensory integration was modulated by attention resources. Similarly, Talsma and Woldorff (2005) required participants to attend to one side (left or right) and detect oddball targets on that side. Event-related potentials (ERPs) results showed an early audiovisual integration around 100 ms for the attended side but not for the unattended side. They also found integration effects at the time window of 160–200 ms and 320–420 ms. Moreover, these integration effects were stronger for attended stimuli than for unattended stimuli. These findings suggest that attention modulates the integration of audiovisual stimuli in multiple stages.

One approach to solve the above debate about the role of attention in multisensory integration is to manipulate the attention resources. Working memory task is one of these tasks for modulating the available attention resources (Zimmer and Macaluso, 2007; Michail and Keil, 2018). Previous studies have testified there exists a close relationship between attention and working memory (Downing, 2000; Botta et al., 2010; Brunetti et al., 2017; Oberauer, 2019). Moreover, Santangelo et al. (2006, 2008b) did not find a more pronounced exogenous orienting effect by multisensory cues than unimodal cues, suggesting that there exists a supramodal spatial attention module that allocates attentional resources towards stimuli from different senses. By using an n-back task as a secondary task, Michail and Keil (2018) found that the integration of non-speech, audiovisual stimuli was enhanced under reduced attentional resources (high WM load condition), suggesting that top-down attentional control plays an essential role in multisensory integration.

Previous studies have demonstrated that the presence of multisensory stimuli could affect working memory. For example, Botta et al. (2011) found that spatially congruent multisensory cues showed a more pronounced attentional effect on working memory as compared to unimodal visual cues. This multisensory advantage remained when multisensory stimuli were used as targets for memorizing (Mastroberardino et al., 2008). These results suggest that multisensory integration can facilitate working memory performance. However, few studies have focused on how working memory affects multisensory integration. Thus, in the present study, to investigate the relationship between attention and multisensory processing, we adopted an n-back working memory task to manipulate the attention resources and explored the multisensory processing under different working memory load conditions.

To date, most studies focused on the multisensory integration of task-relevant stimuli, i.e., multisensory stimuli were used as targets. However, multisensory targets are supposed to capture attention because participants voluntarily allocate attention to them to complete the task. It remains unclear whether multisensory distractors can attract attention more effectively than unisensory distractors and whether attention is needed for the multisensory integration of distractors. Although the multisensory integration of targets has been widely studied in recent two decades, only recently has the multisensory integration of distractors been studied. By using a modified multisensory flanker task (for a review, see Merz et al., 2021), Jensen et al. (2019) and Merz et al. (2019) found that multisensory integration of task-irrelevant stimuli was modulated by overt attention. Specifically, audiovisual and visuotactile distractors were integrated only when they were presented inside the focus of overt attention. In their follow-up study (Jensen et al., 2020), they found that the audiovisual distractors matching the attentional set induced a significant interference effect. By contrast, this interference effect disappeared when the audiovisual distractors did not match the attentional set, indicating that attention was a key factor in the integration of multisensory distractors. Similarly, by using a central visual search task with peripheral distractors, Lunn et al. (2019) did not find significant differences in the interference effects between unisensory (visual) and multisensory (audiovisual) distractors. These findings suggest that multisensory integration does not occur when stimuli are task-irrelevant or not attended to. However, by recording the ERPs, Van der Burg et al. (2011) found an early integration (around 50 ms) of audiovisual distractors. Although the behavioral costs of audiovisual distractors were not significant, this result demonstrated that audiovisual distractors could also be integrated automatically.

The present study aimed to investigate whether multisensory distractors could be integrated and affect attention more effectively than unisensory distractors. Moreover, we explored whether the multisensory integration of audiovisual distractors was modulated by working memory load. In three experiments, 1-back (low load) and 2-back (high load) tasks were adopted. Participants were required to perform the central n-back task while ignoring the peripheral auditory, visual or audiovisual distractors. In Experiment 1, simple white discs and pure tones were used as distractors. To elucidate the neural correlates of the processing of multisensory distractors, Experiment 2 recorded ERPs on the basis of Experiment 1. Given that most of the real-world stimuli contain semantic information and are more complicated compared with simple stimuli, to which extent the results of the simple stimuli can be extended to complex stimuli need to be considered. By using ecological real-life stimuli, previous studies have found that semantic congruence can affect multisensory processing, suggesting the necessity of using semantic real-word stimuli (Mastroberardino et al., 2015; Kvasova et al., 2019; Almadori et al., 2021). Therefore, to improve the ecology of the stimuli, another ERP experiment (Experiment 3) was conducted by adopting pictures of animals and the sounds they made as distractors. We hypothesized that compared with unisensory distractors, multisensory audiovisual distractors were more effective to attract attention and were prone to interfere with the performance of the n-back task. Moreover, the working memory load was expected to modulate this interference effect. That is, the interference effect should be stronger under high load conditions, especially for audiovisual distractors. For the ERPs, the integration effect should be more pronounced in the high load conditions than in the low load conditions. Moreover, the integration of audiovisual distractors should be modulated by the complexity of distractor stimuli, as reflected by the time window of the significant integration effect. That is, the time window of the significant integration effect should be observed later for complex distractors than for simple distractors.

## 2 Experiment 1

### 2.1 Method

#### 2.1.1 Participants

According to the effect size of a similar study [$\eta_{p}^{2}$ = 0.10; Experiment 4 in Lunn et al. (2019)], a sample size estimation was done using G*power software (Faul et al., 2009). The result revealed that a sample of 26 participants was required to at least detect an interaction with an effect size of $\eta_{p}^{2}$ = 0.10 (*α* = 0.05, 1-*β* = 0.80). Twenty-nine healthy college students participated in the experiment. They reported a normal or corrected-to-normal vision and normal hearing. Three participants were excluded because the accuracy rates were lower than 70%. Data of 26 participants (19 females; mean age = 19.92 years, SD = 1.74, range = 18–24 years) entered the final analysis. All participants signed informed consent and were paid 25 RMB. The study was approved by the Ethics Committee of the Department of Psychology, Sun Yat-sen University.

#### 2.1.2 Apparatus and stimuli

The experiment was controlled by E-prime 2.0 software^1^. Participants sat 60 cm in front of a 23-inch monitor (1,920 × 1,080; 60 Hz) in a sound-attenuated, dimly lit room. The auditory stimuli were generated by Adobe Audition CC 2019 software, sampled at 44.1 kHz, and quantized to 16 bits. Before the experiment, the sound was tuned to a comfortable volume for all participants (range: 35–45 dB).

All consonants except “Y” were used (1° × 1.4°of visual angle) in the n-back task. Auditory distractors (1,000 Hz pure tone) were presented for 200 ms either on the left or right side equiprobably *via* headphones (SONY MDR-XB450). Visual distractors were white discs (diameter: 1.9° visual angle), presented at an eccentricity of 7.5° degrees (screen center to discs center). All visual distractors were presented either to the left or right side of the central letters with equal probability for 200 ms. In the audiovisual distractors condition, auditory and visual distractors were presented at the same side concurrently.

#### 2.1.3 Design and procedure

**Figure 1:**
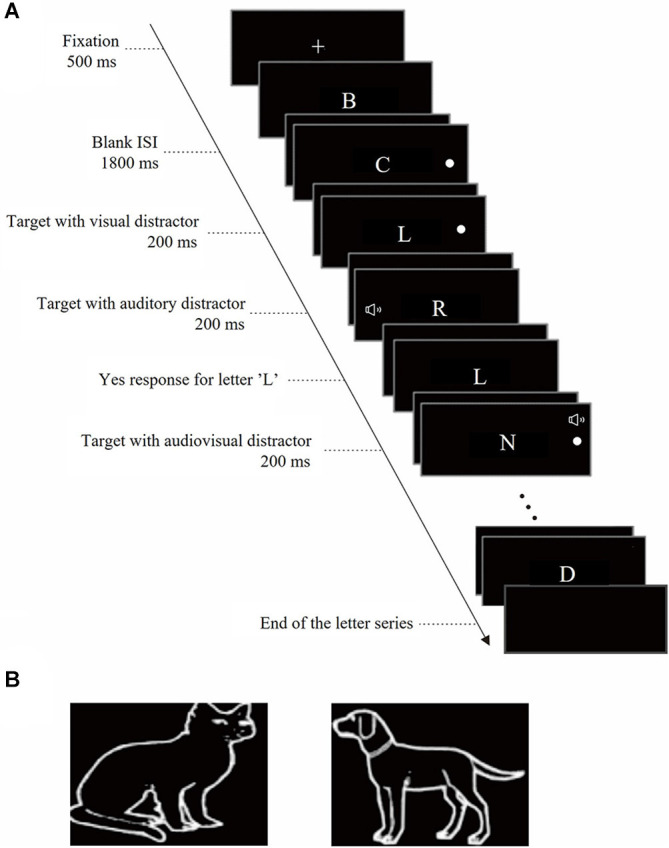
**(A)** Trial scheme in Experiment 1 and Experiment 2 (2-back task in Experiment 1 as an example). **(B)** Stimuli used in Experiment 3.

A 2 (Load: low vs. high) × 4 (Distractor type: auditory, visual, audiovisual vs. no distractor) within-participants design was adopted. The 1-back and 2-back tasks were used to manipulate the working memory load. The trial scheme is shown in Figure 1. Each letter series started with a cross fixation presented at the center of the screen for 500 ms. The fixation was then replaced by a stream of fourteen letters. Each letter was presented for 200 ms with an inter-stimulus interval (ISI) of 1,800 ms. Participants were required to memorize the stream of the letter and to report whether the current target letter was the same or not as the letter presented one or two steps back, in 1-back and 2-back tasks respectively. They were instructed to respond by pressing one of two buttons (LB and RB) with the joystick as quickly and accurately as possible. Response keys on the joystick were counterbalanced between participants.

In addition to the central visual targets, an auditory, visual, or audiovisual distractor was also presented concurrently with the letter in 75% of all trials. They were presented equiprobably at the left or right side of the letter for 200 ms. Participants were required to ignore the peripheral distractor and concentrate on the central letter. In the remaining 25% of all trials, no distractors were presented. Overall, the experiment consisted of 8 blocks with 8 letter series each. Participants had practiced before the formal experiments.

#### 2.1.4 Data analysis

Reaction times (RTs) and accuracy rate (ACC) were calculated separately for each experimental condition. For all participants, RTs of correct responses between 100 and 1,800 ms were included in the analysis. Besides, RTs exceeding ± 3 SD of each participant’s mean reaction time in each experimental condition were removed. Analyses of variance (ANOVAs) were calculated separately for mean RTs and mean ACC with factors of Load (low vs. high) and Distractor type (auditory, visual, audiovisual vs. no distractor). The interference effect was calculated by subtracting the mean RT without distractors from the mean RT with distractors. If necessary, the Greenhouse-Geisser method was adopted to correct degrees of freedom. Besides, Bonferroni corrections were used for *post-hoc* pair-wise comparisons and simple effects.

### 2.2 Results

**Figure 2:**
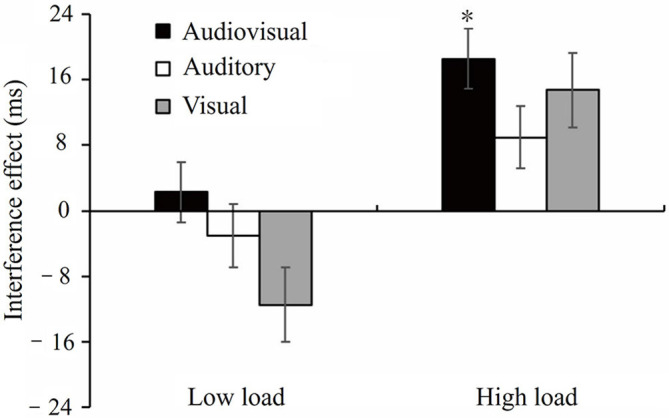
Means and standard errors for the interference effect (RTs with distractors minus RTs without distractors) under auditory, visual, and audiovisual distractors conditions. The interference effect was shown under low and high working memory load, respectively. * indicates *p* < 0.05. RTs, reaction times.

For RTs, the ANOVA revealed a significant main effect of Load [*F*_(1, 25)_ = 155.46, *p* < 0.001, $\eta_{p}^{2}$ = 0.86], suggesting that responses in the low load condition were faster than those in the high load condition (*M* = 574.05 vs. 773.49 ms, SE = 18.01 vs. 29.78). We also found a significant interaction between Load and Distractor type [*F*_(3, 75)_ = 3.51, *p* < 0.05, $\eta_{p}^{2}$ = 0.12; Figure 2]. Follow-up analyses showed that responses to letters with audiovisual distractors were significantly slower than to letters without distractors in the high load condition (*M* = 781.44 vs. 762.94 ms, SE = 30.20 vs. 29.32, *t*_(25)_ = 3.05, *p* < 0.05); whereas in the low load condition, no significant differences were found between these two conditions. No other significant *post-hoc* pair-wise comparisons or main effect [Distractor type: *F*_(2.36, 59.08)_ = 1.98, *p* = 0.14] was found.

For ACC, the ANOVA revealed a significant main effect of Load [*F*_(1, 25)_ = 34.59, *p* < 0.001, $\eta_{p}^{2}$ = 0.58], reflecting that higher ACC was observed in the low load condition than in the high load condition (*M* = 0.95 vs. 0.92, SE = 0.01 vs. 0.01). Neither the main effect of Distractor type [*F*_(2.38, 59.54)_ = 1.55, *p* = 0.22, $\eta_{p}^{2}$ = 0.06] nor the interaction between Load and Distractor type [*F*_(3, 75)_ = 1.57, *p* = 0.20, $\eta_{p}^{2}$ = 0.06] was significant.

### 2.3 Discussion

Overall, we found a significant interference effect of multisensory audiovisual distractors in Experiment 1, as reflected by longer RTs in the working memory task when audiovisual distractors were presented compared to no distractors were presented. However, such a significant interference effect was shown only under high load conditions rather than under low load conditions, suggesting that AV distractors easily draw attention away from the main task under high load conditions compared to the absence of distractors.

It should be noted that a significant response difference between unisensory and multisensory signals does not necessarily mean that the multisensory stimuli have been integrated. According to previous reviews (Stein et al., 2010; Keil and Senkowski, 2018), multisensory integration is defined more strictly as the neural process difference between the unisensory and multisensory stimuli. Thus, to further investigate the neural correlate of the effect of audiovisual distractors, that is, whether the multisensory integration happens or not, we recorded ERPs in Experiment 2.

## 3 Experiment 2

### 3.1 Method

#### 3.1.1 Participants

Based on the effect size of a similar study [$\eta_{p}^{2}$ = 0.10; Experiment 4 in Lunn et al. (2019)], a sample size estimation was done using G*power software (Faul et al., 2009). The result revealed that a sample of 26 participants was required to at least detect an interaction with an effect size of $\eta_{p}^{2}$ = 0.10 (*α* = 0.05, 1-*β* = 0.80). A new group of thirty-two healthy college students participated in the experiment. They had a normal or corrected-to-normal vision and normal hearing. Three participants were excluded because of excessive (>25%) EEG artifacts. Data of 29 participants (16 females; mean age = 20.62 years, SD = 1.99, range = 18–26 years) entered the final analysis. All participants signed informed consent and were paid 75 RMB. The study was approved by the Ethics Committee of the Department of Psychology, Sun Yat-sen University.

#### 3.1.2 Apparatus and stimuli

The experimental apparatus and stimuli were the same as those in Experiment 1 except for the eccentricity of the distractors (4°), and the presenting mode of the auditory stimuli. Specifically, auditory distractors were presented at either the left or right side equiprobably *via* two invisible loudspeakers (Creative inspire T12), which were placed at the source location of the visual distractors behind the screen. Before the experiment, the sound was tuned to a comfortable volume for all participants (range: 65–75 dB).

#### 3.1.3 Design and procedure

A 2 (Load: low vs. high) × 4 (Distractor type: auditory, visual, audiovisual vs. no distractor) within-participants design was adopted. There were 105 trials for each experimental condition. The procedure and task were the same as those in Experiment 1 (Figure 1).

#### 3.1.4 Electroencephalogram (EEG) recording and preprocessing

The EEG was recorded from 64 Ag-AgCl electrodes mounted in an elastic cap (Easy Cap, Germany) with a NeuroScan SynAmps2 Amplifier (Scan 4.5, Neurosoft Labs, Inc. Virginia, USA). A left earlobe electrode was used as an online reference. The ground electrode was located on the forehead. Vertical eye movements were monitored with two electrodes upper and below the right eye. Horizontal eye movements were recorded with two electrodes placed at the outer canthi of each eye. Electrode impedance was kept below 5 kΩ for all electrodes. Online recordings were bandpass filtered at 0.05–100 Hz (12 dB/oct, 40 dB/dec) and sampled at 500 Hz. During the experiment, participants were instructed to fixate on the center of the monitor and try not to make horizontal or vertical eye movements.

The offline analysis of EEG data was performed using Matlab R2016b and eeglab 14.1.2b^2^. First, all scalp electrodes were re-referenced to the average of left and right earlobes. Then, the continuous EEG was bandpass filtered (IIR Butterworth, filter order = 2) at 0.05–30 Hz. An infomax independent component analysis (ICA) algorithm (Bell and Sejnowski, 1995) was applied for correcting eye movement artifacts. The SASICA plugin with ADJUST was used to identify the artifact component. Furthermore, the interval of 0–200 ms prior to the distractors served as the baseline; EEG signal epochs ended 800 ms after the onset of the distractor stimuli, yielding a total epoch of 1 s. Finally, trials with voltages exceeding ± 100 μV were excluded from ERP averages. The remaining epochs to eight different conditions were averaged separately for each participant with baseline corrections. In the present experiment, the average artifact rejection rate was 3.23% of all trials (SD = 5.02, range = 0–18.2%).

#### 3.1.5 Data analysis

Behavioral data analyses were identical to Experiment 1. Analyses of variance (ANOVAs) were calculated separately for mean RTs and mean ACC with within-participants factors of Load (low vs. high) and Distractor type (auditory, visual, audiovisual vs. no distractor).

For the ERPs, to control for the overlap and generic cognitive process (such as contingent negative variation, CNV), the ERPs elicited by no distractor trials were subtracted from the ERPs elicited by auditory (A), visual (V), and audiovisual (AV) distractors, respectively. Then, to estimate the multisensory integration effect, the ERPs elicited by A distractors and V distractors were summated (A + V) and compared with the ERPs elicited by AV distractors. Specifically, the audiovisual distractors were integrated if significant differences were found between the (A + V) ERPs and the AV ERPs (Giard and Peronnet, 1999; Stevenson et al., 2014).

Time windows and electrodes were selected based on the previous studies (Talsma and Woldorff, 2005; Van der Burg et al., 2011), the grand average ERPs and the topographic map. Previous studies have found three phases of effects of integration and/or attention beginning at around 160 ms, and peaking at 190 ms (scalp positivity), 250 ms (negativity), and 300–500 ms (positivity) after stimulus onset (Talsma and Woldorff, 2005). We also did a mass-univariate statistical analysis with corrections based on previous studies. Specifically, we did ANOVAs with factors of Load and Distractor type at each electrode and each time-point across participants. To avoid the type-I error due to the large number of tests, the multisensory integration effects were thought to be significant only when the *p*-value was smaller than 0.05 at 10 (~20 ms) or more continuous time points on at least two nearby electrodes (Van der Burg et al., 2011; Alsius et al., 2014). Two time windows of 240–340 ms (electrode FP_Z_) and 450–600 ms (electrode F_Z_) were selected. To further test the hypothesis whether multisensory integration should be more pronounced in the high load condition than in the low load condition, the mean amplitudes in these time windows were analyzed by ANOVAs with factors of Load (low vs. high) and Distractor type (A + V vs. AV). If necessary, the Greenhouse-Geisser method was adopted to correct degrees of freedom. Besides, Bonferroni corrections were used for *post-hoc* pair-wise comparisons and simple effects.

### 3.2 Results

#### 3.2.1 Behavioral performance

The overall results of ANOVA are shown in Table 1. For RTs, ANOVA revealed a significant main effect of Load [*F*_(1, 28)_ = 52.86, *p* < 0.001, $\eta_{p}^{2}$ = 0.65], indicating slower responses in high load condition than in low load condition (*M* = 790.25 vs. 636.85 ms, SE = 31.97 vs. 22.90). Neither the main effect of Distractor type [*F*_(2.44, 68.37)_ = 0.68, *p* = 0.54] nor the interaction between Load and Distractor type [*F*_(3, 84)_ = 0.30, *p* = 0.83] was significant.

**Table 1:** Results of the ANOVA in Experiments 2 and 3.

	Behavioral results		ERP results	
	RT	ACC	Early integration	Late integration
Experiment 2				
Load	52.86 (<0.001)	17.81 (<0.001)	1.21 (0.28)	3.89 (0.06)
Distractor type	0.68 (0.57)	3.68 (0.02)	0.24 (0.63)	2.19 (0.15)
Load × Distractor type	0.30 (0.83)	0.75 (0.52)	4.57 (0.04)	0.29 (0.59)
Experiment 3				
Load	115.59 (<0.001)	18.88 (<0.001)	0.64 (0.50)	2.13 (0.16)
Distractor type	8.20 (<0.001)	0.57 (0.64)	0.54 (0.47)	2.99 (0.09)
Load × Distractor type	0.24 (0.87)	2.04 (0.11)	0.26 (0.61)	4.69 (0.04)

*F*-value and *p*-value (in parentheses).

For ACC, the main effect of Load was significant [*F*_(1, 28)_ = 17.81, *p* < 0.001, $\eta_{p}^{2}$ = 0.39], indicating ACC in low load condition was higher than in high load condition (*M* = 0.94 vs. 0.90, SEs = 0.01). The main effect of Distractor type was significant [*F*_(3, 84)_ = 3.68, *p* < 0.01, $\eta_{p}^{2}$ = 0.12]. However, *post-hoc* analyses did not reveal any significant pair-wise comparisons. The interaction between Load and Distractor [*F*_(3, 84)_ = 0.75, *p* = 0.52] type was not significant, either.

#### 3.2.2 ERP results: overlap correction

**Figure 3:**
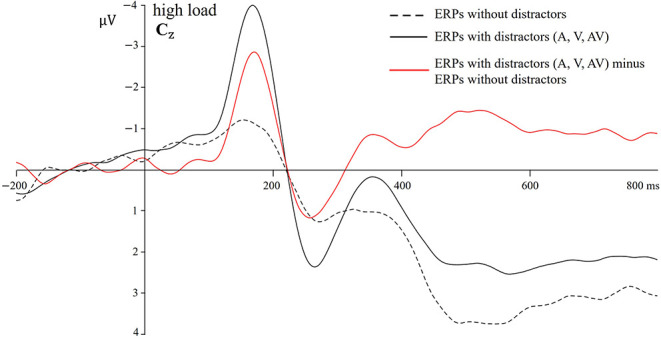
Overlap correction. Averaged ERPs with distractors (averaged with overlaps), ERPs without distractors, and corrected averaged ERPs (averaged ERPs with distractors minus ERPs without distractors) under high load conditions at the C_Z_ electrode. ERPs, event-related potentials.

To control for the differential overlap and generic cognitive process, overlap correction was done before summating the A and V ERPs (Giard and Peronnet, 1999; Talsma and Woldorff, 2005; Van der Burg et al., 2011; Stevenson et al., 2014). To show the necessity of this overlap correction, ERPs elicited by stimuli with A, V, and AV distractors under high load conditions at the C_Z_ electrode were averaged, and then ERPs elicited by stimuli without distractor under high load conditions at the C_Z_ electrode were subtracted from the averaged ERP waveform. The effect of overlap correction was shown in Figure 3.

To further quantify the effectiveness of overlap correction, we tested whether the ERPs elicited by stimuli with (A + V) and AV distractors differed before the onset of the distractor. Theoretically, no significant differences in the mean amplitudes should be found during any time window of −200–0 ms. Besides, the averaged ERPs of distractors should not differ from 0 μV. Thus, the time window of −20–0 ms at the C_Z_ electrode was selected. The one-sample *t*-test was used to test whether the averaged ERPs elicited by stimuli with distractors differed from 0 μV. ANOVA with factors of Load (low vs. high) and Distractor type (A + V vs. AV) was used to test whether the ERPs elicited by stimuli with (A + V) and AV distractors differed from each other. When no-distractor ERPs were not subtracted from ERPs of (A + V) and AV distractors, although no significant results were found for the ANOVA (main effect of Distractor type: [*F*_(1, 28)_ = 1.56, *p* = 0.22, $\eta_{p}^{2}$ =0.05]), the one-sample *t*-test showed that the averaged ERPs with distractors differed significantly from 0 μV [*t*_(28)_ = −5.80, *p* < 0.001; *M* = −0.49 μV, SE = 0.08]. However, after subtracting the no-distractor ERPs from the (A + V) and AV ERPs, neither the *t*-test [*t*_(28)_ = −1.77, *p* = 0.09] nor the main effect of Distractor type [*F*_(1, 28)_ = 0.06, *p* = 0.81, $\eta_{p}^{2}$ = 0.03] was significant. These results showed that subtracting the ERPs elicited by no-distractor trials from the ERPs elicited by A, V, and AV distractor trials could effectively remove the overlap due to the generic cognitive process.

#### 3.2.3 ERP results: early integration

**Figure 4:**
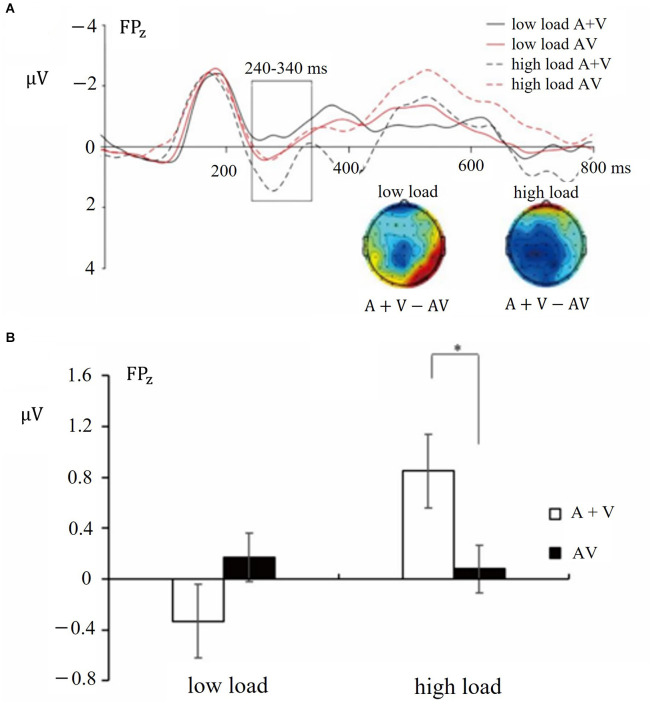
**(A)** Grand-average distractor-synchronized ERP waveforms at each experimental condition. Time window of 240–340 ms is highlighted in the box (electrode FP_Z_). Time zero on the x-axis corresponds to distractor stimuli onset. **(B)** Mean amplitudes and standard errors for different conditions during time window of 240–340 ms (electrode FP_Z_). * indicates *p* < 0.05.

The results of the ANOVA conducted at each time epoch are reported in Table 1. The ANOVA of mean amplitudes for the time window of 240–340 ms showed a significant interaction between Load and Distractor type (*F*_(1, 28)_ = 4.57, *p* < 0.05, $\eta_{p}^{2}$ = 0.14; see Figure 4). Follow-up analyses showed that the mean amplitudes of (A + V) ERPs were more positive than those in the AV condition while the working memory load was high (*M* = 0.85 vs. 0.08 μV, SE = 0.60 vs. 0.41; *t*_(28)_ = 2.10, *p* < 0.05). However, under low load conditions, no significant differences in mean amplitudes were observed between these two conditions. These results showed that audiovisual distractors were integrated under high load conditions but not under low load conditions, suggesting that the working memory load modulated the integration of audiovisual distractors. Neither the main effect of Load [*F*_(1, 28)_ = 1.21, *p* > 0.05] nor the main effect of Distractor type [*F*_(1, 28)_ = 0.24, *p* > 0.05] was significant.

In addition, the ANOVAs of mean amplitudes for the late time window 450–600 ms showed no significant interaction between Load and Distractor type [*F*_(1, 28)_ = 0.29, *p* > 0.05]. Neither the main effect of Load [*F*_(1, 28)_ = 3.89, *p* > 0.05] nor the main effect of Distractor type [*F*_(1, 28)_ = 2.19, *p* > 0.05] was significant.

### 3.3 Discussion

By adopting the ERP technique, we investigated whether audiovisual distractors could be integrated and how working memory load affected the multisensory integration. Although no significant interactions were found in behavioral performances, ERP results showed that working memory load modulated the integration of audiovisual distractors, that is, the audiovisual distractors could be integrated only under high load conditions. Specifically, under high load conditions, after the overlap correction, significant differences in the ERP mean amplitudes were found between the (A + V) distractor conditions and the AV distractor conditions. This finding indicated that audiovisual distractors were integrated under high working memory load conditions, while such a pattern was eliminated under low load condition. The results in Experiment 2 are in line with the findings in Experiment 1, that is, the capacity of inhibiting distractors is reduced under high load condition.

Experiment 2 demonstrated that the integration of simple audiovisual distractors happened at the time window of 240–340 ms after the onset of the distractors, indicating an early integration of audiovisual distractors under high load conditions. This time window of audiovisual integration is consistent with previous studies focusing on the integration of audiovisual targets (Teder-Sälejärvi et al., 2002; Talsma and Woldorff, 2005; Van der Burg et al., 2011). For instance, Teder-Sälejärvi adopted a multisensory oddball paradigm and found integration at the time window of 242–226 ms and 300–400 ms after the onset of the audiovisual targets.

The distractors used in Experiments 1 and 2 are simple stimuli (visual discs and auditory pure tones). However, most of the stimuli we interact with in real life are complex stimuli, and to which extent the results of the integration of simple stimuli can be extended to complex stimuli should be considered (Koelewijn et al., 2010). Thus, in Experiment 3, by adopting animal pictures and the sounds they made as distractors, we further investigated how the multisensory audiovisual distractor affected attention.

## 4 Experiment 3

### 4.1 Method

#### 4.1.1 Participants

In reference to the effect size of a similar study [$\eta_{p}^{2}$ = 0.10; Experiment 4 in Lunn et al. (2019)], a sample size estimation was done using G*power software (Faul et al., 2009). The result revealed that a sample of 26 participants was required to at least detect an interaction with an effect size of $\eta_{p}^{2}$ = 0.10 (*α* = 0.05, 1-*β* = 0.80). Another new group of thirty-seven healthy college students participated in the experiment. They had a normal or corrected-to-normal vision and normal hearing. Two participants were excluded because of equipment problems. Another three participants were excluded because of excessive (>25%) EEG artifacts. Data of 32 participants (21 females; mean age = 20.19 years, SD = 2.15, range = 18–27 years) entered the final analysis. Participants signed informed consent and were paid 75 RMB. The study was approved by the Ethics Committee of the Department of Psychology, Sun Yat-sen University.

#### 4.1.2 Apparatus and stimuli

The experimental apparatus and stimuli were the same as those in Experiment 2 except for the type of distractors. Specifically, visual distractors consisted of pictures of a cat or a dog. They were presented for 500 ms at an eccentricity of 4° degrees (screen center to image center). Auditory distractors were the sounds that the animals made. All auditory stimuli were presented at either the left or right side equiprobably for 500 ms *via* two invisible loudspeakers (Creative inspire T12) placed at the source location of the visual distractors behind the screen. In the multisensory audiovisual distractors condition, both auditory and visual distractors of the same animal were presented on the same side concurrently. Before the experiment, the sound was tuned to a comfortable volume for all participants (range: 65–75 dB).

#### 4.1.3 Design and procedure

A 2 (Load: low vs. high) × 4 (Distractor type: auditory, visual, audiovisual vs. no distractor) within-participants design was adopted. There were 105 trials for each experimental condition. In order to convey semantic information clearly, central letters and peripheral distractors were both presented for 500 ms. The stimuli used as distractors are shown in Figure 1B. The procedure and task were the same as those in Experiment 1.

#### 4.1.4 Electroencephalogram (EEG) recording and preprocessing

EEG recording and preprocessing were identical to Experiment 2. The average artifact rejection rate in the present experiment was 2.27% of all trials (SD = 4.19, range = 0–20.4%). ERPs in each experimental condition were averaged separately for each participant.

#### 4.1.5 Data analysis

Behavioral data analyses were identical to Experiment 2. Analyses of variance (ANOVAs) were calculated separately for mean RTs and mean ACC with within-participants factors of Load (low vs. high) and Distractor type (auditory, visual, audiovisual vs. no distractor).

For the ERPs, the data analyses were identical to Experiment 2. After the overlap correction, time windows and electrodes were selected based on the previous studies (Talsma and Woldorff, 2005; Van der Burg et al., 2011), the grand average ERPs and the topographic map. We did a mass-univariate statistical analysis (ANOVAs with factors of Load and Distractor type at each electrode and each time-point across participants) with correction based on previous studies. That is, the multisensory integration effects were thought to be significant only when the *p*-value was smaller than 0.05 at 10 (~20 ms) or more continuous time points on at least two nearby electrodes (Van der Burg et al., 2011; Alsius et al., 2014). Two time windows of 250–330 ms (averaged across electrodes C_Z_ and FC_Z_) and 440–600 ms (averaged across electrodes AF7, F5, and F7) were selected. To further test the hypothesis whether multisensory integration should be more pronounced in the high load condition than in the low load condition, the mean amplitudes in these two time windows were analyzed by ANOVAs with factors of Load (low vs. high) and Distractor type (A + V vs. AV), respectively. If necessary, Greenhouse-Geisser method was adopted to correct degrees of freedom. Besides, Bonferroni corrections were used for *post-hoc* pair-wise comparisons and simple effects.

### 4.2 Results

#### 4.2.1 Behavioral performance

The overall results of ANOVA are shown in Table 1. For RTs, ANOVA revealed a significant main effect of Load [*F*_(1, 31)_ = 115.59, *p* < 0.001, $\eta_{p}^{2}$ = 0.79], indicating the responses were slower in the high load condition than in the low load condition (*M* = 700.72 vs. 562.29 ms, SE = 26.62 vs. 17.92). The main effect of Distractor type was significant [*F*_(3, 93)_ = 8.20, *p* < 0.001, $\eta_{p}^{2}$ = 0.21], indicating the responses to letters with visual distractors (*M* = 639.35 ms, SE = 21.79) were slower than to letters with auditory, audiovisual distractors and with no distractors (auditory: *M* = 625.15 ms, SE = 21.76; audiovisual: *M* = 630.29 ms, SE = 21.80; no distractors: *M* = 631.22 ms, SE = 21.98). The interaction between Load and Distractor Type was not significant [*F*_(3, 93)_ = 0.24, *p* = 0.87, $\eta_{p}^{2}$ = 0.01].

For ACC, there was a significant main effect of Load [*F*_(1, 31)_ = 18.88, *p* < 0.001, $\eta_{p}^{2}$ = 0.38], indicating that ACC in the low load condition was higher than in the high load condition (*M* = 0.94 vs. 0.89, SEs = 0.01). Neither the main effect of Distractor type [*F*_(3, 93)_ = 0.57, *p* = 0.64, $\eta_{p}^{2}$ = 0.02] nor the interaction [*F*_(2, 93)_ = 2.04, *p* = 0.11, $\eta_{p}^{2}$ = 0.06] was significant.

#### 4.2.2 ERP results: overlap correction

Similar to Experiment 2, ERPs elicited by stimuli with A, V, and AV distractors under high load conditions at the C_Z_ electrode were averaged, and then ERPs elicited by stimuli without distractors in the high load condition at the C_Z_ electrode were subtracted from the averaged ERP waveform.

To further quantify the effectiveness of overlap correction, we tested whether the ERPs elicited by stimuli with (A + V) and AV distractors differed before the onset of the distractor. The time window of −20–0 ms at the C_Z_ electrode was selected. The one-sample *t*-test was used to test whether the averaged ERPs elicited by stimuli with distractors differed from 0 μV. The two-way ANOVA with factors of Load (low vs. high) and Distractor type (A + V vs. AV) was used to test whether the ERPs elicited by stimuli with (A + V) and AV distractors differed from each other. When no-distractor ERPs were not subtracted from ERPs of (A + V) and AV distractors, the one-sample *t*-test showed that the averaged ERPs with distractors differed significantly from 0 μV [*t*_(31)_ = −5.33, *p* < 0.001; *M* = −0.51 μV, SE = 0.10]. The ANOVA also showed a significant main effect of Distractor type [*F*_(1, 31)_ = 7.12, *p* < 0.05, $\eta_{p}^{2}$ = 0.19], indicating more negative ERPs elicited by stimuli with (A + V) distractors than with AV distractors (*M* = −0.95 vs. −0.49 μV, SE = 0.22 vs. 0.10; *t*_(31)_ = 2.66, *p* < 0.05). However, after subtracting the no-distractor ERPs from the (A + V) and AV ERPs, neither the *t*-test [*t*_(31)_ = 0.12, *p* = 0.90] nor the main effect of Distractor type [*F*_(1, 31)_ = 0.18, *p* = 0.67, $\eta_{p}^{2}$ = 0.01] was significant. These results showed that subtracting the ERPs elicited by no-distractor trials from the ERPs elicited by A, V, and AV distractor trials effectively removed the overlap due to the generic cognitive process.

#### 4.2.3 ERP results: late integration

**Figure 5:**
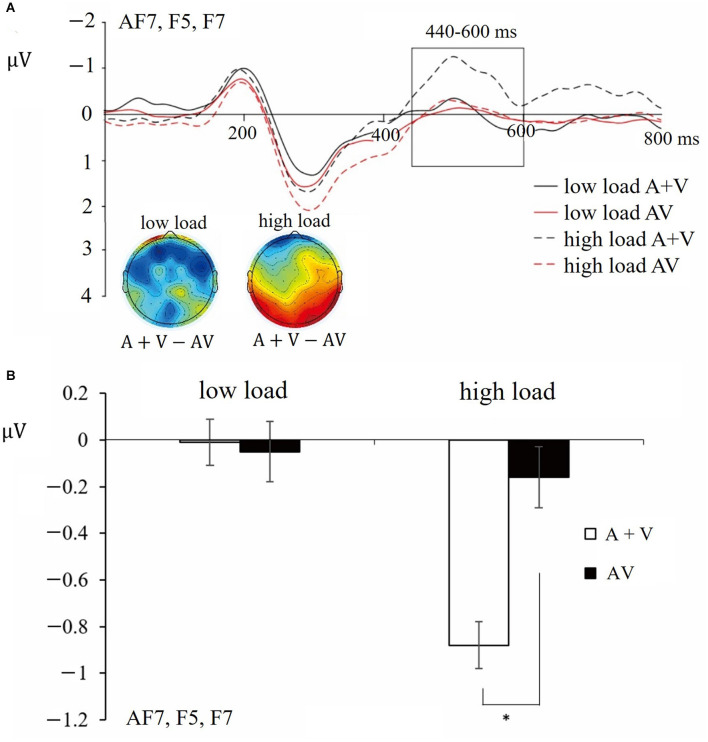
**(A)** Grand-average distractor-synchronized ERP waveforms at each experimental condition. Time window of 440–600 ms is highlighted in the box (electrodes AF7, F5, F7). Time zero on the x-axis corresponds to the distractor stimulus onset. **(B)** Mean amplitudes and standard errors for different conditions during time window of 440–600 ms (electrodes AF7, F5, F7). * indicates p < 0.05.

The results of the ANOVA conducted at each time epoch are reported in Table 1. The ANOVA of mean amplitudes of 440–600 ms showed a significant interaction between Load and Distractor type [*F*_(1, 31)_ = 4.69, *p* < 0.05, $\eta_{p}^{2}$ = 0.13; see Figure 5]. Follow-up analyses showed that the mean amplitudes of (A + V) ERPs were more negative than those in the AV condition when the working memory load was high (*M* = −0.88 vs. −0.16 μV, SE = 0.38 vs. 0.25; *t*_(31)_ = 2.67, *p* < 0.05). However, under low load conditions, no significant differences in mean amplitudes were observed between these two conditions. Similarly, as in Experiment 2, the present results showed that audiovisual distractors were integrated under high load conditions but not under low load conditions. Moreover, the working memory load modulated the integration of audiovisual distractors at the late stage when the audiovisual distractors were the meaningful complex stimuli. Neither the main effect of Load [*F*_(1, 31)_ = 2.13, *p* > 0.05] nor the main effect of Distractor type [*F*_(1, 31)_ = 2.99, *p* > 0.05] was significant.

In addition, the ANOVAs of mean amplitudes of the early time window (250–330 ms) showed no significant interaction between Load and Distractor type [*F*_(1, 31)_ = 0.26, *p* > 0.05]. Neither the main effect of Load [*F*_(1, 31)_ = 0.46, *p* > 0.05] nor the main effect of Distractor type [*F*_(1, 31)_ = 0.54, *p* > 0.05] was significant.

### 4.3 Discussion

By adopting the ERP technique, the present results echo the main findings in Experiment 2. That is, the working memory load modulated the integration of audiovisual distractors. Specifically, audiovisual distractors could be integrated only under high load conditions. This was demonstrated by the significant interaction between Load and Distractor type, indicating there existed a late integration of AV distractors at the time window of 440–600 ms over frontal electrodes.

Moreover, the complex distractor stimuli used in the present study were more ecological than the simple stimuli. As expected, a relative late integration effect of audiovisual distractors for complex stimuli was found, which was reflected by the late time window of significant integration effect in ERPs. This late integration is consistent with the findings in previous studies using complex audiovisual stimuli (Raij et al., 2010; Xi et al., 2020). For instance, Xi et al. (2020) adopted pictures and sounds of animals and inanimate objects as targets and asked participants to perform a discrimination task, in which participants had to attend to one side (left or right) and only respond to the target stimuli at that side. They found three late effects of semantic integration, that is, the time window of 220–240 ms and 560–600 ms for attended stimuli and the time window of 340–360 ms for unattended stimuli. Together with the previous studies, our present results support that compared to the simple stimuli, it takes more time to integrate complex stimuli and thus a later time window of integration in ERPs was observed.

## 5 General discussion

The present study aimed to investigate the effect of working memory load on the processing of audiovisual distractors. We measured the behavioral and electrophysiological responses to central letters with auditory, visual, and audiovisual distractors under different working memory loads. Through three experiments, we demonstrated that multisensory (audiovisual) distractors could effectively interfere with the n-back task, especially under high working memory load conditions. Moreover, the time course of the integration of audiovisual distractors depends on the complexity of distractor stimuli. That is, the integration of the audiovisual distractors is reflected as an early integration (240–340 ms) of simple distractors and a late integration (440–600 ms) of complex distractors.

First of all, behavioral performance in Experiment 1 showed slower responses to letters with peripheral audiovisual distractors than to letters without distractors, which was significant only under high working memory load condition. This result demonstrated that high working memory load strengthened the interference effects of audiovisual distractors. That is, audiovisual distractors were effective to interfere with the performance (e.g., slowing down the responses) in the main working memory task under high load condition. Similarly, our ERP results further supported this finding. In both Experiment 2 and Experiment 3, mean amplitudes were more pronounced for stimuli with (A + V) distractors than stimuli with AV distractors under high load conditions. By contrast, under low load conditions, no significant differences in mean amplitude were observed between these two conditions. These results indicate that working memory load modulates the integration of audiovisual distractors, which is consistent with previous results (de Fockert et al., 2001). That is, in the low load condition, participants have enough attention resources to select and process task-relevant stimuli and inhibit the processing of distractors. However, in the high load condition, attention capacity is overloaded, leaving fewer resources to inhibit the distractors. Thus, the distractors disengage attention from the working memory task more easily under high load condition, i.e., showing the interference effect. These results suggest that compared to unisensory distractors, audiovisual distractors can be more effective in disengaging attention from the main working memory task, and the processing of audiovisual distractors is modulated by the availability of attention resources.

Moreover, ERPs results revealed both an early and a late integration of audiovisual distractors under high working memory load condition. Specifically, the integration of simple audiovisual distractors happened at the time window of 240–340 ms after the onset of the distractors in Experiment 2, while a late integration of complex audiovisual distractors was found at the time window of 440–600 ms in Experiment 3. Similar to Experiment 1, the integration of audiovisual distractors was only found under high load condition, suggesting that integration of audiovisual distractors needs top-down attention control. This finding is consistent with the integration framework of one early review (Koelewijn et al., 2010). That is, unimodal inputs are processed independently in each modality and are then integrated at a late stage. Moreover, the different time courses of the multisensory integration of simple and complex distractors found in the current study might reflect the distinct awareness and processing of distractor stimuli. Compared with simple audiovisual distractors, the integration of complex audiovisual distractors may require semantic processing and is time-consuming, resulting in a late time window of integration. For instance, Xie et al. (2017) adopted a delayed matching-to-sample task, in which participants were required to judge whether the probe stimulus (visual) was the same as the target stimulus (visual, auditory, or audiovisual). The stimuli they used consisted of line drawings of real-life objects and the sound they made, such as animals, tools, vehicles, etc. They found a relatively late semantic target integration at the time window of 236–530 ms, which was due to the requirement of top-down processing for the integration of semantic information.

Our study can broaden the understanding of the role of attention in multisensory integration. Previous studies in this field mainly focused on the crossmodal integration of targets (Santangelo and Spence, 2007; Zimmer and Macaluso, 2007). However, multisensory targets are supposed to be easily attended to and responded to. Thus, it is worth investigating the integration of multisensory distractors besides the targets, which can help us better understand whether attention is needed for multisensory integration. Here, we found that attention could modulate the audiovisual integration at both early and late stages, which is consistent with previous studies (Michail and Keil, 2018; Lunn et al., 2019) and frameworks trying to resolve the inconsistent results on the relationship between attention and multisensory integration (Koelewijn et al., 2010; Navarra et al., 2010; Talsma et al., 2010). Researchers have considered key factors that modulate multisensory integration, such as stimuli complexity, stimuli competition, perceptual load, etc. Specifically, when the stimuli are complex, or the cognitive load is high, the current goal determines which stimuli are integrated first (top-down attentional control). When the stimuli are simple or the cognitive load is low, the stimuli could be integrated automatically (bottom-up processing without attention). Therefore, our results further demonstrate the importance of working memory load for the integration of audiovisual distractors.

It should be noted that although we found a significant interaction between Load and Distractor type in Experiment 1, i.e., significant interference effect of multisensory distractors under high WM load condition, such a behavioral result was not shown in Experiments 2 and 3. This inconsistency might be due to the slight difference across the experimental setting. On one hand, 7.5 degrees of visual angle was used for distractors in Experiment 1, while in Experiments 2 and 3, distractors were present at 4 degrees of visual angle. Previous studies have found that distractors could cause more interference at a peripheral location than at a central location (Chen, 2008; Corral and Escera, 2008), resulting in a decreased behavioral effect in both Experiments 2 and 3. On the other hand, a headphone was used in Experiment 1, while loudspeakers were adopted in Experiments 2 and 3. In Experiment 1, the audio was presented *via* headphones to the left or right ear of the participants. In Experiments 2 and 3, audio was presented *via* loudspeakers placed at the same location as the video behind the screen. Therefore, spatial (left or right side) information of audio in Experiments 2 and 3 was not as accurate as in Experiment 1, which might also reduce the interference effect of distractors. One previous study has reported a significant interference effect by peripheral sound distractors when using headphones but not using loudspeakers (Corral and Escera, 2008). Nevertheless, we found significant multisensory integration effects in the ERP results of Experiments 2 and 3. Maybe the integration happened, but it was not strong enough to be observed at the behavioral level. For example, a previous study also used audiovisual distractors and found a significant early integration (around 50 ms) in ERP results but fail to observe the behavioral cost (Van der Burg et al., 2011). Using unisensory and multisensory cues, Santangelo et al. (2008b) also revealed no increase orienting effect following bimodal as compared to unimodal cues, while the ERPs elicited by bimodal cues were more pronounced than the sum of the ERPs elicited by unisensory cues. These results suggest multisensory integration can happen even without observing behavioral benefits. In addition, the integration effect of distractors may not be as strong as that of targets shown in previous studies. Nevertheless, as the first evidence investigating the integration of distractors, we provide its cognitive and neural mechanisms by using the ERP method.

In conclusion, compared to unisensory auditory or visual distractors, multisensory audiovisual distractors can disengage participants’ attention more effectively, thus observing significant interference effects for audiovisual distractors. Moreover, working memory load modulates the processing of audiovisual distractors. Only under high load condition do the audiovisual distractors disengage attention from the working memory task and interfere with the task performance effectively. Our results support that attention is necessary for the occurrence of multisensory integration. Moreover, the integration of simple audiovisual distractors occurs at an early stage (240–340 ms), while a late integration stage (440–600 ms) for complex audiovisual distractors.

## Data availability statement

The datasets presented in this study can be found in online repositories. The names of the repository/repositories and accession number(s) can be found below: https://osf.io/wh273.

## Ethics statement

The studies involving human participants were reviewed and approved and this study was approved by the Ethics Committee of Department of Psychology, Sun Yat-sen University (2020-0325-0127). The patients/participants provided their written informed consent to participate in this study.

## Author contributions

YY and XH: conceptualization, data curation, formal analysis, investigation, methodology, resources, software, validation, visualization, writing—original draft, writing—review and editing. ZY: conceptualization, funding acquisition, methodology, project administration, resources, supervision, writing—original draft, writing—review and editing. All authors contributed to the article and approved the submitted version.

## Footnotes

^1^ http://www.pstnet.com/

^2^ https://www.mathworks.com/
